# Printed Graphene, Nanotubes and Silver Electrodes Comparison for Textile and Structural Electronics Applications

**DOI:** 10.3390/s21124038

**Published:** 2021-06-11

**Authors:** Agnieszka Tabaczyńska, Anna Dąbrowska, Marcin Słoma

**Affiliations:** 1 OPTEX S.A., Oskara Kolberga 2, 26-300 Opoczno, Poland; 2 Department of Personal Protective Equipment, Central Institute for Labour Protection–National Research Institute, Wierzbowa 48, 90-133 Lodz, Poland; andab@ciop.lodz.pl; 3 Institute of Metrology and Biomedical Engineering, Faculty of Mechatronics, Warsaw University of Technology, Św. Andrzeja Boboli 8, 02-525 Warsaw, Poland; marcin.sloma@pw.com.pl

**Keywords:** textile electronics, printed and structural electronics, electrically conductive textiles, wearable electronics, graphene, nanoparticles, composites

## Abstract

Due to the appearance of smart textiles and wearable electronics, the need for electro-conductive textiles and electro-conductive paths on textiles has become clear. In this article the results of a test of developed textile electro-conductive paths obtained by applying the method of screen printing pastes containing silver nanoparticles and carbon (graphene, nanotubes, graphite) are presented. Conducted research included analysis of the adhesion test, as well as evaluation of the surface resistance before and after the washing and bending cycles. Obtained results indicated that the samples with the content of carbon nanotubes 3% by weight in PMMA on substrate made of aramid fibers (surface mass of 260 g/m^2^) were characterized by the best adhesion and the best resistance to washing and bending cycles. Such electro-conductive paths have potential to be used in smart clothing applications.

## 1. Introduction

The industry of smart textiles, sometimes called active, interactive and adaptive textiles (smart and intelligent textiles and clothing) [1,2,3] incorporated in garments, has been extensively developed. Its history started back in the middle of last century when the first heated clothing was developed, followed by the discovery of shape-memory materials; these together marked a new era of innovations in materials. These inventions were followed some decades ago by the application of these materials for textile products, introducing to the market smart textiles [4]. Further developing these ideas, researchers started to work on the combination of smart and conductive textiles with electronic components. 

This way, intelligent wearable textile systems with embedded electrically conductive materials were created and called e-textiles [2,5,6,7,8]. Application challenges for e-textiles have been summarized by Wang et al. [9]. Authors indicated that due to their closeness to the human body, higher demands are placed on them such as low weight, small size and flexibility which are usually failed by 2D and 3D electronic devices. Komolafe et al. [10] additionally indicates that e-textiles should be durable and should keep the traditional textile properties that make them appropriate for clothing applications. Therefore, researchers look for the solutions in which electrically conductive fibrous structures are introduced into the textile product without causing a visual change [8]. 

Special attention is paid to the development of e-textiles for monitoring of physical and physiological parameters of human body that will not negatively influence user comfort [11,12,13,14]. Therefore, in the few last years, several projects (e.g., i-Protect, PROETEX, WearIT, My Heart, BioTex, Stella, Context, Ofseth) aimed at the development of smart clothing with the function of monitoring the user and/or the environment were carried out within the national and European framework programs. 

From a textile fabrication process point of view, the challenge is to make as many connections and active or passive components as possible coated or embedded in the textile material. Therefore, to develop electro-conductive textiles several approaches are proposed, for example by integrating conductive fibres or yarns, applying conductive coatings, or using conductive printable inks [4]. Leśnikowski [15] analysed the possibility to develop microstrip and coplanar textile signal lines with characteristic impedance not higher than 50 ohms, and proposed a solution in which electro-conductive fabric was sewn onto a non-conductive substrate. Moreover, he showed [16] that the influence of humidity on the impedence of the textile signal lines depends on the substrate material. A use of lock-stitch embroidery technique for the purpose of preparation of conductive tracks in fabricating e-textiles was considered by Zheng et al. [17]. Their research was focused on embroidery made of silver-coated polyamide yarn on the knitted fabric. On the basis of the obtained results, authors indicated embroidery parameters that may be used for conductive tracks for smart clothing. 

The application potential of inkjet printing for manufacturing of electronic devices such as solar panels, sensors, and transistors was indicated by Beedasy [18]. Authors analysed state of the art in this field of electronics’ inkjet printing, indicating glass, paper and polymers as most common substrates. Among the polymers, mainly polyimide (PI), polyethylene terephthalate (PET), polyethylene napthalate (PEN) and polydimethylsiloxane (PDMS) are mentioned. Shahariar et al. [19] emphasized that inkjet printing on textile substrate is a novel approach and very challenging in terms of ensuring required functionality with a use of this technology for the purpose of wearable electronic textiles. As the main issues to overcome, the following challenges are mentioned: blocking nozzles and achieving electrically conductive percolation on rough textile substrate. In the paper, authors presented the inkjet printing process on three kinds of substrates: knitted, woven and non-woven fabrics. The lowest sheet resistance was obtained in the case of polyester woven fabric which was equal to 0.2 Ω/sq, which predestines this as a solution for e-textile applications. Karim et al. [20] highlighted that inkjet printing for manufacturing of electro conductive fabrics is a very promising technique due to its excellent processing and environmental benefits, while limited ink stability and cost related to electro-conductive material (e.g., Ag) concentration in the ink are its bottlenecks. Authors have overcome those issues by means of optimization of composite and combination of a silver nanoparticle-based ink with a highly concentrated graphene dispersion. Stempien et al. [21] also noticed an issue of ink stability, which is particularly important in the case of textile substrates due to their roughness. Therefore, authors proposed a water-soluble ink with silver content together with simultaneous sintering at 90 °C, and when eight layers were printed on different fabrics (made of polyacrylonitrile, polypropylene, polyester and basalt fibres) a surface resistance at the level of 0.155 0.235 Ω/sq was achieved. Conductive inks are used in many applications, including electronics, computers and communications. These rigid substrates of PCBs (Printed Circuit Boards) are different from textiles; therefore different technologies have to be applied, such as screen printing [4]. 

The aim of this study is to improve the functional characteristics of intelligent clothing by replacing the rigid electrical connections (between sensors, measuring and control devices and transmission systems) with the developed flexible textile material, with highly electrically conductive paths, resistant to maintenance cycles. In this study, screen printing techniques have been used for flame-retardant textile materials to achieve the effect of electro-conductivity by covering them with electro-conductive pastes based on silver, graphene and carbon nanotubes. The use of screen printing techniques to provide electro-conductive paths on fabrics made of either aramid or modacrylic fibers that provides resistance to thermal factors of the clothing is a novelty in the proposed approach. 

According to the performed analysis of the state-of-the-art, up to now, only a few experiments considered a use of a screen printing method on flame-retardant fabrics. It is worth mentioning that this is an extremely important aspect in terms of potential applications in protective clothing for fire fighters. At the same time, the influence of the kind of textile substrate (defined by e.g., manufacturing technique, wave, square weight, raw material composition) on electrical conductivity was confirmed. An attempt to make an electro-conductive textile material by a screen printing method with the use of aramid fabric was made by Kazani [22] with a low surface resistance reaching 0.020–0.048 Ω/□ (one of the lowest values from the samples considered by Kazani) depending on the ink used. However, in this work only woven aramid fabric was used, and after 20 washing cycles the surface resistance of those samples increased to 11–36 Ω/□. Therefore, on the basis of these results it can be concluded that the use of this method is promising in terms of application into the flame retardant protective clothing; however, further research works are required in order to improve resistance to washing cycles. Moreover, taking into account the multi-layer structure of protective clothing for fire fighters, including a non-woven flame-retardant layer, the use of such a textile structure for screen printing with electro-conductive ink seems to be a novel research direction worth consideration. This technique seems to be also more appropriate for applications in protective clothing for fire fighters than embroidery due to the fact that in the case of embroidery, the electro-conductive thread goes through the fabric and as a consequence may transfer heat from the external environment close to the user’s body. Positive results of the conducted research can highly contribute to further progress in e-textiles for protective clothing applications. 

## 2. Materials and Methods

### 2.1. Materials

#### 2.1.1. Textile Materials

In this study, three variants of woven textile substrates were selected. Their characteristics are presented in Table 1.

#### 2.1.2. Electrically Conductive Composites

For the electrically conductive paths application of composite coatings in the form of a paste consisting of a polymer vehicle carrier and the functional phase were selected. The detailed description of the paste composition of the composites is described below.

As a base polymer material, polymethacrylates (PMMA) and fluorovinylidene (PVDF) materials were used, previously applied for the printing of electronic circuits on elastic foils or glass substrates [14]. They were selected by the criteria of easy processing and low cost, which allows for the production of low-cost printed electronic circuits. These polymers allow the formation of composite pastes with a rheology suitable for applying layers with the screen printing technique. The resulting layers should have a specific electrical properties (sheet resistivity/resistance) and acceptable mechanical properties, allowing them to be flexible.

In the study the following polymers were used:

- organic vehicle in the form of poly (methyl-methacrylate) (PMMA) having a molecular weight of 350,000 mol supplied by Sigma-Aldrich dissolved in butyl carbitol acetate (OKB) with a concentration of 8 wt.%;
- LuxPrint DuPont 8155 resin (available commercially), intended for the manufacturing of composite pastes for screen printing, in particular for the compositions comprising metal powders and ceramics. The main component of the fluoroelastomer is a vinylidene (PVDF) dissolved in ethyl acetate cellosolve (OCE).

For the functional phase, the following electrically conductive particles were selected: silver particles, graphite, graphene platelets, multi-walled carbon nanotubes. Commercially available multi-walled carbon nanotubes (MWCNTs, CNTs) from CheapTubes were used, while the graphene platelets (GNPs) were supplied and prepared at the Institute of Electronic Materials Technology (ITME) in Warsaw. In the study, two types of the following composite pastes were used:

- commercially available composite paste with silver particles (L-121, from Institute of Electronics Materials Technology, Warsaw, Poland) and graphite paste (421 electrodag SS, Acheson),
- developed pastes containing graphene and carbon nanotubes.

Composite with silver conductive phase pastes contained commercially available silver flakes AX20LC (Amepox) with 99% Ag purity, a mean flake size of 2–4 µm.

**Figure 1:**
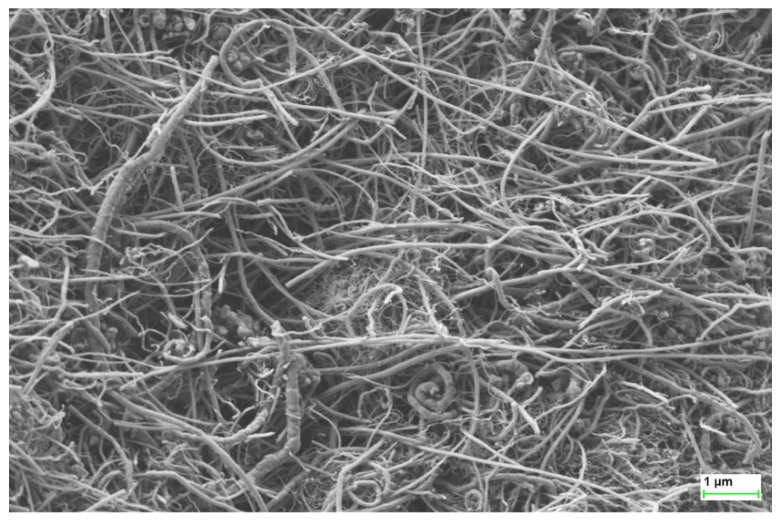
Scanning Electron Microscopy micrograph image of used multi-walled carbon nanotubes-AURIGA Cross Beam Workstation (Carl Zeiss).

Figure 1 shows an image taken using SEM (Scanning Electron Microscope), presenting the characteristic dimensions of multi-walled carbon nanotubes used in the research. The mean values of the characteristic dimensions are: diameter (10 ÷ 50) nm, a length of 0.5 µm to 15 µm.

**Figure 2:**
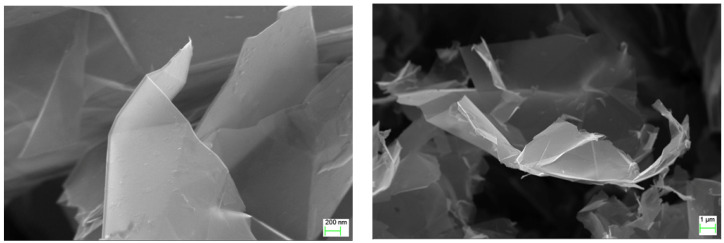
Scanning Electron Microscopy micrograph image of used graphene platelets-AURIGA Cross Beam Workstation (Carl Zeiss).

Graphene flakes were prepared in ITME with direct exfoliation process. The base material in form of graphite flakes (180–300 µm) was added to the surfactant, and deionized water. Then, the processes of intercalation expansion, exfoliation, freezing and freeze drying and separation of graphene flakes was carried out. Figure 2 presents the results of SEM analysis. Electrical properties of graphene flakes are as follows:

- resistance R_s_ = 0081 Ω/□
- conductivity δ = 115,525 S/m
- carrier mobility 172 cm^2^/V∙s
- carrier concentration −6.618 × 10^17^ cm^−2^

Obtained graphene nanoplatelets characteristic dimensions are: diameter approx. 15 microns thickness of less than 20 layers.

#### 2.1.3. Printed Samples Preparation

In the study, the following marking of the samples were applied:

- X-the Roman numeral indicating the variant with respect to the chemical composition of the paste (I to XV)
- Y-Arabic number means a series of produced pastes (for the series in this study it is always 2),
- Z-Arabic number indicates one of three textile materials used as the substrate (e.g., 1, 2, 3).

Below in Table 2, the sample characteristics of used variants of pastes are presented.

Prepared composite pastes were deposited on the textile substrates with the screen-printing technique on an AMI Presco 242 screen-printer with 67T mesh polyester screens. The printed layers were cured at 120 °C for one hour in a laboratory oven dryer.

**Figure 3:**
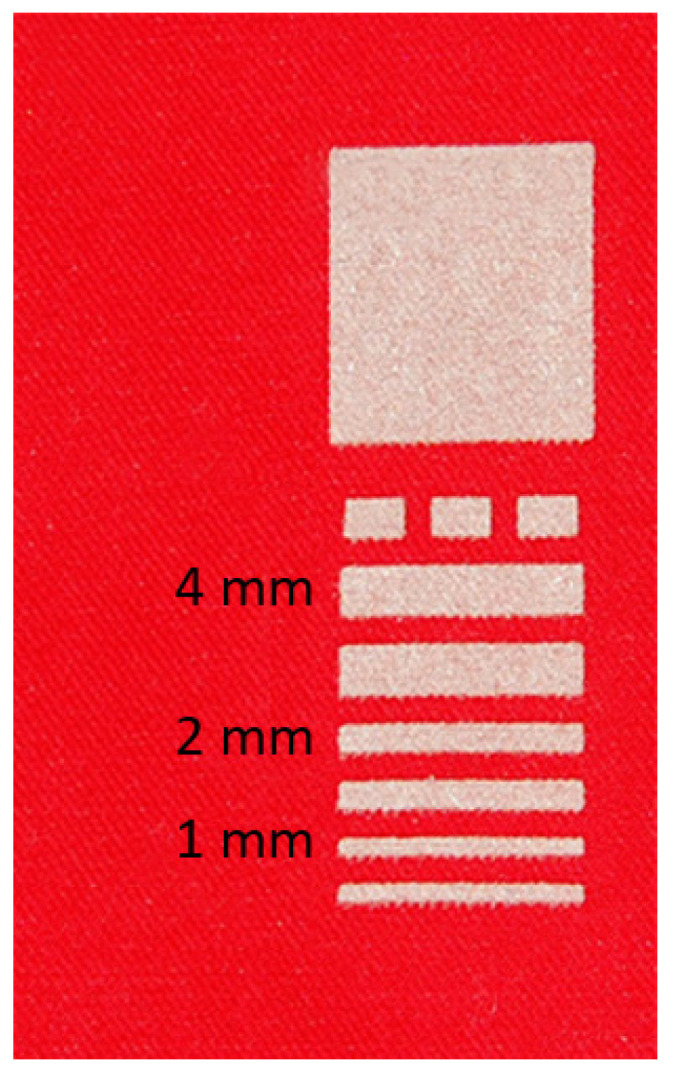
General view of the topology of electro-conductive printed paths for all prepared samples–here silver conductive paths.

**Figure 4:**
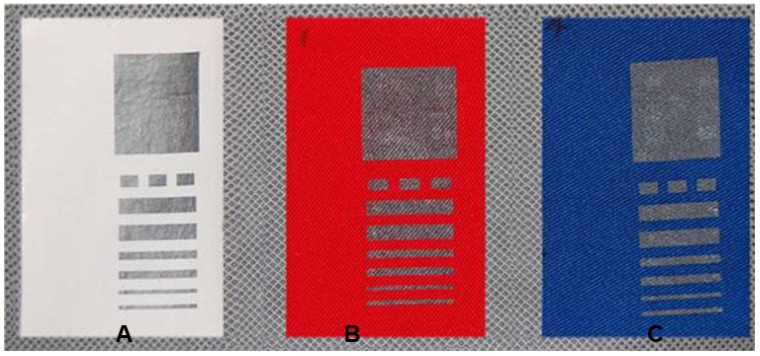
Views of the samples covered with paste of the composition of carbon nanotubes XIV 1 wt.% PVDF OCE three fabrics: (**A**): material 1, (**B**): material 2, (**C**): material 3.

Each of the three selected textile materials was covered by fifteen variants of pastes with different composition described in Table 2 (designated I-XV) in the model form shown in Figure 3 and Figure 4. As an example designation, IV. 2.1 means a sample where, on the textile no. 1 (non-woven fabric laminated film PTFE), a paste with a composition of 5 wt.% of graphene platelets in PMMA was applied. Due to the structure of the fabrics it is very hard to measure the thickness of printed layers. However, the thickness of the printed layers can be estimated to be around 10 μm, as observed for the layers printed with the same screen on a rigid, flat substrate.

### 2.2. Test Methods

For the evaluation of developed paths, the following laboratory tests were performed: adhesion tests and surface resistance of new material, as well as after washing and bending cycles.

#### 2.2.1. Adhesion Test

For the evaluation of the adhesion of the printed layer to the fabric, the scotch tape test was performed. The tape was adhered to the surface of the sample and pressed with a finger, then allowed to stabilize for five minutes. In the next step the tape was torn from the surface at an angle of 180° with two speeds: v_1_ = 20 mm/s and v_2_ = 40 mm/s. The amount of deposited material on the surface of the adhesive tape, as well as the defects in the printed paths, were assessed. To evaluate the adhesion, a five-stage scale of 0–4 was used. The test method of rating adhesion by the tape test is described in ASTM D3359 [23].

#### 2.2.2. Surface Resistance

For each sample, the surface resistance of the printed path was measured. Measurements were made using a laboratory digital multimeter Agilent 34401Ain an ambient temperature of 21°C and relative humidity of 45%. Due to the different width of electrically conductive paths, measurements were performed for each path and then the appropriate conversion to sheet resistance was applied, with respect to the number of geometry squares in the measured topology (sheet resistance is expressed in Ω/□ or Ω/sq units). Conversion to the sheet resistance is the most common approach in characterising electrical properties of conductive thin and thick films and other planar systems. The value of sheet resistance is not related to the scaling of the planar geometry, allowing the comparison of the electrical properties of different size layers and electrodes. The value of sheet resistance is calculated with the formula:

(1) $$R=\frac{\rho }{t}\frac{L}{W}=R_{s}\frac{L}{W}$$

where *R* is measured resistance, *ρ* is bulk resistivity, t is thickness of the layer, *L* is length, and *W* is width. This way, having measured resistance and knowing the planar geometry of the sample (length and width), we can calculate uniform sheet resistance by one square surface of the layer, regardless of the actual length and width of the measured sample:

(2) $$R_{s}=R\frac{W}{L}$$

This approach eliminates the element of layer thickness, which is especially problematic to measure and estimate for the conductive lines printed on wavy textile substrate.

For each sample, five measurements were performed. Then, the results for each sample were averaged, and the final result was the lowest resistance value for the measured samples. Differences in the resistance due to width of the electro-conductive paths were also taken into account.

#### 2.2.3. Surface Resistance after Washing and Bending Cycles

In order to assess how maintenance cycles affect the properties of the electrically conductive paths printed samples were subjected to the 10 washing cycles. Washing was performed in accordance with EN ISO 6330: 2012 [24]. Washing was performed by hand at 30 °C using a detergent containing no phosphate, no optical brightener and without enzymes.

**Figure 5:**
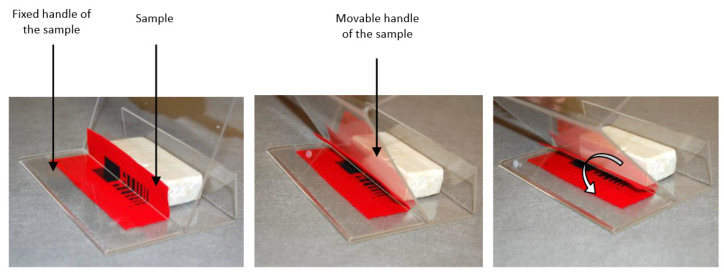
Views on the mechanical bending cycles at different stages of sample’s bending.

The obtained samples were also subjected to a series of bending cycles with the following number of cycles: 10, 20, 30, 50. After each series, the surface resistance was measured. Bending cycles were performed by means of specially designed device presented in Figure 5.

Final influence of the washing and mechanical bending cycles and was evaluated comparing the final value of resistance (*R*_k_) with the initial value of resistance after printing (*R*_0_). The relative change in resistance was determined with the Equation:

(3) $$\Delta R=\frac{R_{0}-R_{k}}{R_{0}}\times 100\%$$

## 3. Results

### 3.1. Adhesion Test Results

Results of the adhesion tests are presented in Table 3, Table 4 and Table 5. The higher the value of the adhesion index, the worse the adhesion of the application to the substrate.

The study showed (Table 3) that the applications of pastes I and II containing the silver particles in PMMA and PVDF respectively on the three textile substrates are resistant to tearing at slow rate of v_1_ = 20 mm/s. Lesser adhesion was observed for the samples evaluated at the speed v_2_ = 40 mm/s. The worst results of the adhesion test were observed for the pastes printed on substrate 1, having a smooth surface of PTFE film. For the graphite paste, the worst adhesion properties were also demonstrated for substrate 1 (adhesion value 4). For the other substrates (2 and 3) adhesion value was acceptable (0 and 1).

The study showed (Table 4) that the use of nanofillers in the form of multi-walled carbon nanotubes, 1 wt.%, 3 wt.% and 5 wt.%, respectively, does not have any negative influence on the adhesion to the fabric substrate 2 and 3, and it is comparable with the results obtained for the pastes with the silver particles printed on substrate 1.

In the study (Table 5) it was observed that the use of 5 wt.%, 7 wt.% and 10 wt.%. of the graphene nanoplatelets in the paste showed inferior adhesion to the textile substrate in relation to the nanofiller in the form of nanotubes. In this case, there is also a tendency of higher adhesion to the textile substrate 2 and 3 than to the PTFE coated film on substrate 1.

The best adhesion was observed for the pastes with nanotubes in PMMA and PVDF vehicles on substrates 2 and 3.

### 3.2. Surface Resistance Results

**Figure 6:**
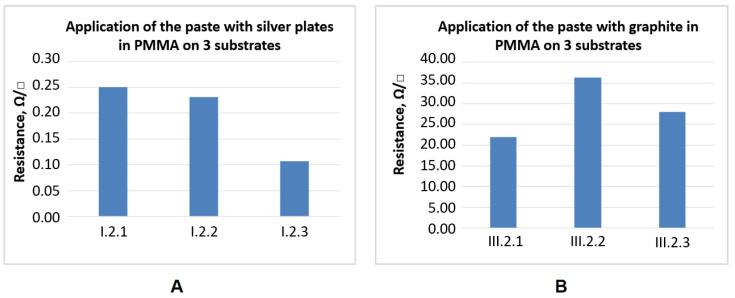
Results of the surface resistance measurements for pastes containing: (**A**)–silver microflakes and (**B**)–graphite microflakes, both in PMMA vehicle on three types of textile substrates.

In the Figure 6 the results of measurements of the surface resistance for the pastes containing silver particles (I) and graphite (III) are presented. Pastes with the silver particles (I) are treated as the reference samples for the developed paste containing carbon nanotubes, and graphene.

The resistance of the paths (Figure 6) printed from pastes with silver in PMMA vehicle on substrate 1 (non-woven) is 0.25 Ω/□ compared with 0.11 Ω/□ on substrate 3 (the woven fabric of aramid fibers). For the pastes with graphite, the values are 22 Ω/□ for substrate 1 (nonwoven with PTFE membrane) and 28 Ω/□ for substrate 3 (aramid fabric), respectively. These results represent the samples made with the use of commercially available paste, therefore, they can be treated as a reference. Obtained results suggest that the paste with silver plates allowed for obtaining much lower surface resistance than in the case of the paste with graphite. 

**Figure 7:**
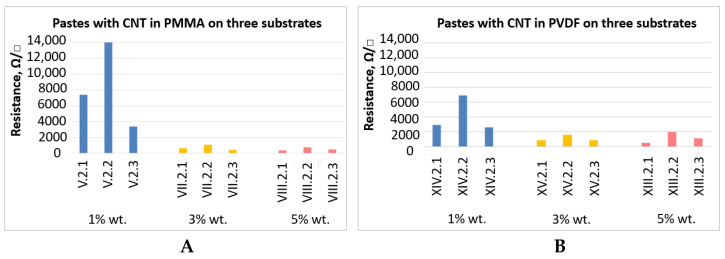
Test results of surface resistance measurements for the pastes with different wt.% content of carbon nanotubes (CNT) in: (**A**)–PMMA and (**B**)–PVDF vehicles, printed on substrates 1, 2 and 3.

In the Figure 7 the values of resistance measurements results for the pastes with different content of carbon nanotubes in PMMA and PVDF are presented.

**Figure 8:**
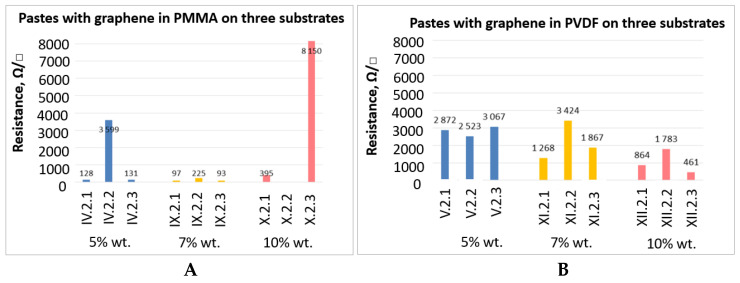
Test results of surface resistance measurements for the pastes with different wt.% content of graphene nanoplatelets in **A**–PMMA and **B**–PVDF, on substrates 1, 2 and 3.

In Figure 8, the values of resistance measurement results for the pastes with different content of graphene nanoplatelets in PMMA and PVDF are presented.

**Figure 9:**
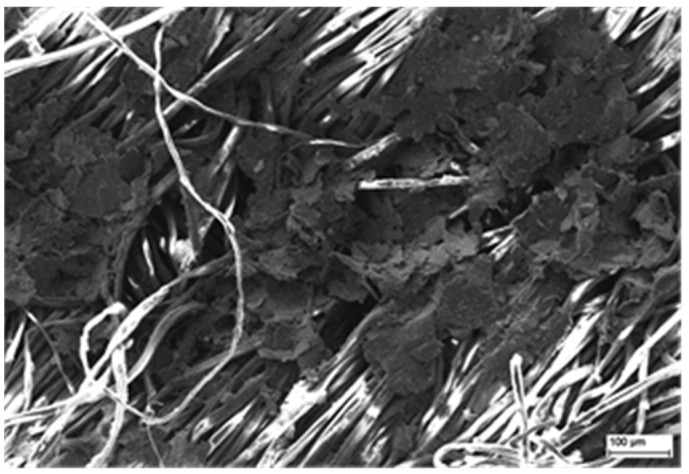
Scanning Electron Microscopy micrograph image presenting the morphology of the sample surface with printed graphene paste containing 10 wt.% GNP in PMMA organic vehicle.

In general, almost all variants of the pastes noted their highest value of surface resistance for substrate 2. This phenomenon is observed under SEM inspections (Figure 9), where we can see the uneven distribution of the paste and its discontinuity on the surface of the fabrics. This is due to the highly uneven surface of substrate textile 2, which has the highest surface mass of the three substrates used, with the highest differences in the texture of the material. Such geometry of the material causes agglomeration of the applied paste in the hollow spaces between the weave of the fabric and creates its local discontinuity of printed paths in the peeks of the weave of the fabric. The unusual results related to the local increase of the sheet resistivity for the composite pastes with higher concentration of conductive filler is due to the higher concentration of the filler resulting in higher viscosity of the paste, which might result in uneven printing on the wavy textile substrate, creating local discontinuities in the layer or reducing its thickness.

### 3.3. Surface Resistance after Washing and Bending Cycles

The aim of the study was to simulate the use of materials with electrically conductive tracks. The greater the index of change in resistance, the worse its resistance to either washing or bending of the electro-conductive path. For most variants a significant influence of washing and bending cycles was observed, therefore, only selected results are presented. 

On the basis of the performed measurements, it was indicated that sample VII. 2.3 (with 3% wt. nanotubes in PMMA) showed the lowest rate (about 15%) of resistance changes after the washing cycles. These results are coherent with observation that the sample VII. 2.3 is characterized by the best adhesion of the paste to the substrate from the samples with 5% nanotube content. For the rest of the samples, the observed change in surface resistance was shaping between 25%—for the samples with 1% content of the nanotubes in PMMA to 50%—for the samples with 5% content of the nanotubes in PMMA. In the case of the samples with the 5%, 7% and 10% content of the graphene nanoplates, a higher rate of change of the surface resistance was observed, which exceeded 50%. 

**Figure 10:**
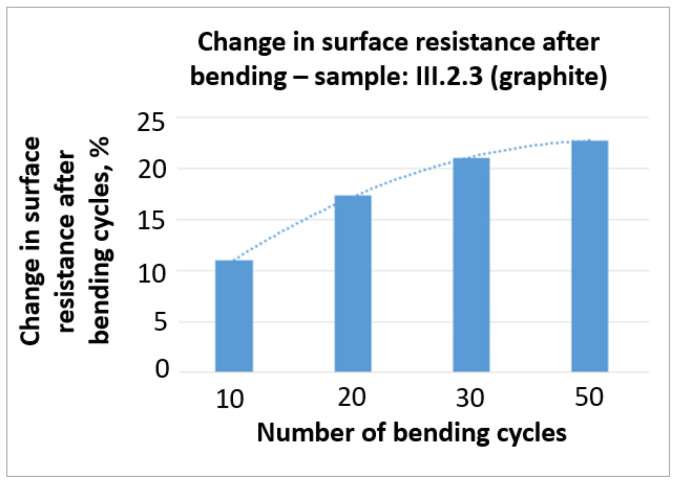
Test results of the change in surface resistance after bending cycles–III. 2.3.

**Figure 11:**
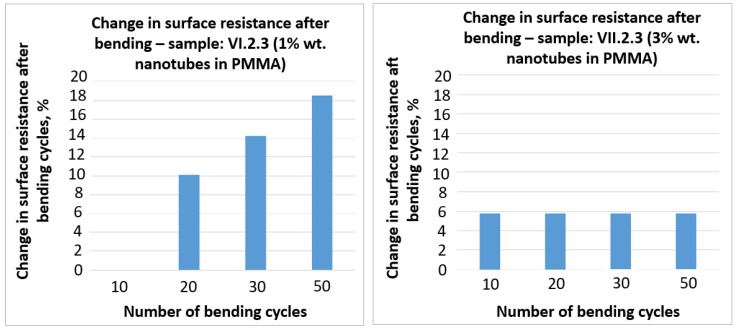
Test results of the change in surface resistance after bending cycles for the pastes with different wt.% content of nanotubes in PMMA in A–1% wt. and B–3% wt., on substrate 3.

The results of the changes in surface resistance of the selected samples are presented in Figure 10 and Figure 11. 

On the basis of the obtained results (Figure 10 and Figure 11), it can be stated that bending has a negative influence on the surface resistance of the tested samples. In the case of the III. 2.3 sample with graphite the change in surface resistance after 50 bending cycles reached more than 20%. A similar result (close to 20%) was obtained in the case of VI. 2.3 sample with 1% wt. nanotubes in PMMA. The lowest change in surface resistance, that in addition was stable with further bending cycles, was obtained in the case of VII. 2.3 with 3% wt. nanotubes in PMMA and did not exceed 6%.

## 4. Discussion

In the studies it was found that the PMMA vehicle allows for obtaining layers with a lower surface resistance than PVDF vehicle for pastes containing a comparable content of graphene nanoplatelets. An exception is the paste with 10 wt.% of graphene in PMMA printed on substrate 3. For the paste based on PVDF, a decrease in the value of the surface resistance with increasing content of graphene nanoplatelets was observed. At the same time, for pastes based on PMMA a decrease in the surface resistance with graphene content of 7 wt.% relative to the 5 wt.% was observed, with an unexpected increase in the value of resistance for 10 wt.% of graphene also observed. This is due to the higher viscosity of the 10 wt.% pastes negatively influencing the printed paths’ uniformity, and hence the conclusion is that for the paste with the graphene nanoplatelets the content of 7 wt.% is most preferred.

Analyzing the results of resistance measurements of pastes with CNTs on substrates 1 and 3 it is noted that, except for the case with the content of 1 wt.% carbon nanotubes in the PMMA polymer, nanotubes in PMMA allow obtaining a lower surface resistance than the PVDF pastes with a comparable content of nanotubes. Application potential of screen-printed electro-conductive fabrics with CNTs was confirmed e.g., by Krucińska et al. [13]. Increasing the content of nanotubes in the polymer from 1 wt.% to 3 wt.% caused a decrease in resistance, with a very small increase of resistance for the 5 wt.% samples–also due to the higher viscosity negatively influencing the printing process, as observed for GNPs. It was found that the lowest resistance was obtained for substrate 3 and pastes with 3 wt.% of nanotubes in PMMA vehicle and on substrate 1 and pastes with 5 wt.% of nanotubes in PMMA vehicle, respectively. This result is in coherence with results obtained e.g., by Kazani et al. [1] who also analyzed the influence of various textile substrate on the surface resistance of the samples made with a use of the screen printing technique. 

The observed unusual increase of the sheet resistivity for the composite pastes with a higher concentration of carbon nanotubes (5 wt.%) and graphene nanoplatelets (10 wt.%) needs additional explanation. While a higher concentration of conductive filler should decrease the sheet resistance, it is at the same time negatively affecting the printing process by increasing the viscosity of the pastes. This results in uneven printing on the wavy textile substrate, creating local discontinuities in the layer or reducing its effective thickness. Such unfavourable phenomenon can be eliminated but needs additional steps in materials preparation. Such an approach should be focused on the optimisation of the paste composition. The addition of viscosity modifiers or dispersing agents should influence the viscosity of the paste, tailoring the rheology for the printing process, even with a higher amount of nanofiller. Obtained this way, printed layers should be uniform and continuous even when printed on high roughness substrates, such as textiles.

The analysis of the final results of the surface resistance measurements after washing cycles led to the conclusion that the best results were obtained for the pastes with CNTs in PMMA vehicle on substrate 3. For the sample with 3 wt.% of CNTs in PMMA on substrate 3, the lowest value of resistance change after washing cycles was observed which was equal to 15%. The obtained results correspond to the observations that the same paste has the best adhesion to the substrate. For the remaining samples, the results were 25% for 1 wt.% and 50% for 5 wt.% of the carbon nanotubes in PMMA, respectively. Our results confirmed that the resistance of the screen-printed electro-conductive layer to washing cycles is limited and requires further processing in order to avoid increasing of the surface resistance after such maintenance processing. This problem was observed also by other authors who look for protecting the electro-conductive layer by application of additional PU coating [1,22].

For most of the samples exposed to bending cycles, the change of the value of coefficient of resistance after 10, 20, 30 and 50 cycles was high. For pastes with silver particles in the PMMA vehicle on three substrates, the values of resistance changed from 50% after 10 cycles to 85% after 50 cycles, respectively. These results indicate that this type of paste with silver particles on the textile substrates is not resistant to the regular use in textronics application in clothing, despite the lowest resistance just after printing. For pastes with graphite, the worst results of the surface resistance on samples before the pre-treatment were obtained for pastes applied on substrate 2, but at the same time the lowest change in resistance after 50 cycles of bending was observed for the paste with graphite applied on this substrate (about 23%). For pastes with graphene nanoplatelets the coefficient of resistance changes were obtained from 10% up to 100% in the case of pastes with PVDF vehicle. In further research, a higher number of bending cycles should be considered.

## 5. Conclusions

In the study, the developed electrically conductive paths printed on three types of textile substrates were presented. The following conclusions summarize our observations:

- Geometry and the type of substrate affect the adhesion of the paste. The poorest adhesion to the substrate was observed for the smooth Teflon (PTFE) coating. The best adhesion was observed for the multi-walled carbon nanotubes in PMMA and PVDF vehicles on the substrates made of modacrylic (substrate 2) and aramid (substrate 3) fibers;
- The more complex the substrate surface, the higher the values of surface resistance observed. It was also found that the highest resistance was observed for substrate 2 (made of modacrylic fibers with a surface mass of 320 g/m^2^) with the most developed surface;
- For substrates 1 (non-woven fabric, laminated with a PTFE membrane) and 3 (aramid fibers), PMMA vehicle allows for obtaining layers with the lowest surface resistance, compared with PVDF for pastes of analogical content of graphene nanoplatelets;
- For the PMMA-based pastes we observed a decrease in the resistance with an increase of the graphene content from 7 wt.% to the 5 wt.%, followed by the increase in the resistance for 10 wt.% samples, due to the higher viscosity of the 10 wt.% pastes influencing negatively the printed paths uniformity;
- PMMA vehicle allows to obtain layers with a lower surface resistance than PVDF vehicle based pastes with comparable content of multi-walled carbon nanotubes;
- The pastes containing multi-walled carbon nanotubes obtained the lowest resistance on substrate 3 (aramid fibers) with 3 wt.% of CNTs in PMMA vehicle, followed by paths printed on substrate 1 (nonwoven fabric and laminated with a PTFE membrane) with 5 wt.% of CNTs in PMMA.

Summarizing the obtained results, it was shown that the samples that are characterized by the best adhesion and the best resistance to washing and bending cycles were the samples with the content of the carbon nanotubes 3% by weight in PMMA on substrate 3 (of aramid fibers, of a weight of 260 g/m^2^). Such materials can be effectively applicable in the advanced textronics applications, withstanding regular wearing and service conditions. The applications of printed paths on textile substrates also go beyond textronics applications, allowing the preparation of pre-pregs for epoxy-fiber composites, therefore creating embedded structural electronics systems. Such composite structures can be adapted for automotive, aerospace and other advanced applications with embedded intelligent sensing, monitoring, heating and other implementations.

## Figures and Tables

**Table 1:** Characteristics of textile materials used as a substrate in the study.

No.	Fabric Composition	Surface Mass, g/m^2^ Thickness, mm (±5%)
1	Non-woven made of aramid and paraaramid fibers (Kevlar/Nomex) laminated with PTFE membrane (Teflon) (white colored yellow).	140 g/m^2^ 0.78 mm
2	Fabric made of modacrylic fand cotton fibers Composition: 54% Protex MA, 46% CO (color red)	320 g/m^2^ 0.60 mm
3	Fabric made of paraaramid/aramid fibers (Nomex Comfort fabric Composition: 93% Nomex, Kevlar 7%) (colored blue).	260 g/m^2^ 0.52 mm

**Table 2:** Characteristics of the samples.

Variant of Chemical Composition of the Paste	The Percentage of Functional Phase in the Paste	Polymer Vehicle Paste
I	Silver flakes	34% PMMA in OKB, paste L121 (from ITME)
II	Silver flakes	PVDF in OCE
III	Graphite flakes	Commercially available paste Electrodag 421 SS(Acheson) E421SS
IV	Graphen nanoplates GNP 5 wt.%	PMMA in OKB
V	Graphen nanoplates GNP 5 wt.%	PVDF in OCE
VI	Multi-walled carbon nanotubes MWCNT 1 wt.%	PMMA in OKB
VII	Multi-walled carbon nanotubes MWCNT 3 wt.%	PMMA in OKB
VIII	Multi-walled carbon nanotubes MWCNT 5 wt.%	PMMA in OKB
IX	Graphen nanoplates GNP 7 wt.%	PMMA in OKB
X	Graphen nanoplates GNP 10 wt.%	PMMA in OKB
XI	Graphen nanoplates GNP 7 wt.%	PVDF in OCE
XII	Graphen nanoplates GNP 10 wt.%	PVDF in OCE
XIII	Multi-walled carbon nanotubes MWCNT 5 wt.%	PVDF in OCE
XIV	Multi-walled carbon nanotubes MWCNT 1 wt.%	PVDF in OCE
XV	Multi-walled carbon nanotubes MWCNT 3 wt.%	PVDF in OCE

**Table 3:** Results of the adhesion test of samples with electro-conductive paste I (with silver in PMMA), II (with silver in PVDF) and III (with graphite in PMMA) on three textile substrates.

No. of Sample	I. 2.1	I. 2.2	I. 2.3	II. 2.1	II. 2.2	II. 2.3	III. 2.1	III. 2.2	III. 2.3
	PMMA			PVDF			PMMA		
v_1_	0	0	0	0	0	0	4	0	0
v_2_	4	2	2	4	1	1	4	1	1

**Table 4:** Results of the adhesion test of samples with electro-conductive paste with nanotubes content in PMMA and PVDF on three textile substrates.

MWNT Nanotubes Content in Polymer	PMMA			PVDF		
	No. of Sample	Adhesion		No. of Sample	Adhesion	
		v_1_	v_2_		v_1_	v_2_
1%	VI. 2.1	0	4	XIV. 2.1	1	4
	VI. 2.2	0	0	XIV. 2.2	0	0
	VI. 2.3	0	1	XIV. 2.3	0	0
3%	VII. 2.1	4	4	XV. 2.1	4	4
	VII. 2.2	1	1	XV. 2.2	0	1
	VII. 2.3	1	1	XV. 2.3	0	1
5%	VIII. 2.1	4	4	XV. 2.1	3	3
	VIII. 2.2	1	1	XV. 2.2	1	2
	VIII. 2.3	1	1	XV. 2.3	1	2

**Table 5:** Results of the adhesion test of samples with electro-conductive paste with graphene flakes content in PMMA and PVDF on three textile substrates.

Graphene Nanoplates Content in Polymer	PMMA			PVDF		
	No. of Sample	Adhesion		No. of Sample	Adhesion	
		v_1_	v_2_		v_1_	v_2_
5%	IV. 2.1	1	3	V. 2.1	3	3
	IV. 2.2	3	3	V. 2.2	2	2
	IV. 2.3	3	3	V. 2.3	2	3
7%	IX. 2.1	2	3	IX. 2.1	3	3
	IX. 2.2	2	3	IX. 2.2	1	2
	IX. 2.3	2	2	IX. 2.3	2	3
10%	X. 2.1	3	3	XII. 2.1	3	3
	X. 2.2	2	2	XII. 2.2	1	2
	X. 2.3	2	2	XII. 2.3	2	2

## Data Availability

The data presented in this study are available on request from the corresponding author.
